# A Facile Microwave Hydrothermal Synthesis of ZnFe_2_O_4_/rGO Nanocomposites for Supercapacitor Electrodes

**DOI:** 10.3390/nano13061034

**Published:** 2023-03-13

**Authors:** Xiaoyao Mo, Guangxu Xu, Xiaochan Kang, Hang Yin, Xiaochen Cui, Yuling Zhao, Jianmin Zhang, Jie Tang, Fengyun Wang

**Affiliations:** 1 College of Physics, Qingdao University, No. 308 Ningxia Road, Qingdao 266071, China; 2 College of Mechanical and Electrical Engineering, Qingdao University, No. 308 Ningxia Road, Qingdao 266071, China; 3 State Key Laboratory of Bio Fibers and Eco Textiles, Qingdao University, No. 308 Ningxia Road, Qingdao 266071, China; 4 National Institute for Materials Science, 1-2-1 Sengen, Tsukuba 305-0047, Japan

**Keywords:** microwave hydrothermal, reduced graphene oxide, ZnFe_2_O_4_ nanoparticles, supercapacitors

## Abstract

As a typical binary transition metal oxide, ZnFe_2_O_4_ has attracted considerable attention for supercapacitor electrodes due to its high theoretical specific capacitance. However, the reported synthesis processes of ZnFe_2_O_4_ are complicated and ZnFe_2_O_4_ nanoparticles are easily agglomerated, leading to poor cycle life and unfavorable capacity. Herein, a facile microwave hydrothermal process was used to prepare ZnFe_2_O_4_/reduced graphene oxide (rGO) nanocomposites in this work. The influence of rGO content on the morphology, structure, and electrochemical performance of ZnFe_2_O_4_/rGO nanocomposites was systematically investigated. Due to the uniform distribution of ZnFe_2_O_4_ nanoparticles on the rGO surface and the high specific surface area and rich pore structures, the as-prepared ZnFe_2_O_4_/rGO electrode with 44.3 wt.% rGO content exhibits a high specific capacitance of 628 F g^−1^ and long cycle life of 89% retention over 2500 cycles at 1 A g^−1^. This work provides a new process for synthesizing binary transition metal oxide and developing a new strategy for realizing high-performance composites for supercapacitor electrodes.

## 1. Introduction

Due to the severe worldwide energy crisis and global environmental degradation, it is necessary to explore renewable and clean energy sources in combination with finding suitable energy storage devices [1] among various energy storage devices, such as secondary batteries, fuel cells, and supercapacitors. Compared with secondary batteries and fuel cells, supercapacitors (SCs) have gained significant attention in recent years owing to their high power density, quick charge/discharge capability, and long cycle life in recent years [2,3,4,5].

Supercapacitors can be divided into electrical double-layer capacitors (EDLCs) and pseudocapacitors arising from different energy storage mechanisms. The EDLCs store charges by electrostatic adsorption/desorption at the interface of the electrodes/electrolytes. Activated carbon, carbon aerogels, graphene, and carbon nanotubes (CNTs) are widely used in EDLCs due to their high specific surface area and high conductivity [6,7,8]. The researchers prepared an activated carbon as a supercapacitor electrode, and it exhibits a high specific capacitance of 333.8 F g^−1^ and long cycle life of 96% retention over 38,000 cycles [8]. Although EDLCs have high power density and long cycle life due to the physical adsorption/desorption, while the low specific capacitance and energy density severely limits their broader application [9,10]. Different from EDLCs, pseudocapacitors usually store charges by fast and reversible surface or near-surface redox reactions [11]. Conducting polymers and transition metal oxides are often used as electrodes of pseudocapacitors, which possess high theoretical specific capacitance [12,13]. However, the undesirable aggregation or volume expansion and poor electrical conductivity of such conducting polymers and transition metal oxides during the charge and discharge process leads to low specific capacitance, poor rate capability, and poor cycle life which limits their practical applications.

Compared with single transition metal oxides, binary transition metal oxides usually possess better electrical conductivity, a different redox potential, and a high specific surface area, which is beneficial for realizing high electrochemical performance [14,15,16]. For example, Zate et al. synthesized Ni_x_Mn_1-x_Fe_2_O_4_ film and used it as the electrode of a supercapacitor which showed a specific capacitance of 185 F g^−1^ at 5 mV s^−1^ for Ni_0.__8_Mn_0.__2_Fe_2_O_4_ [17]. Giri et al. prepared nitrogen-doped rGO–NiMnO_3_ by a hydrothermal method, which delivered a specific capacitance of 749.2 F g^−1^ at 0.5 A g^−1^ [18]. The above results demonstrate the feasibility of binary transition metal oxides as electrode materials for supercapacitors.

As a typical binary transition metal oxide, ZnFe_2_O_4_ gained much attention due to its high theoretical specific capacitance (~2600 F g^−1^), but is still restricted by poor conductivity and severe agglomeration during charge and discharge process, causing low specific capacitance and cycle life [19,20]. Numerous studies have confirmed that synthesizing composites with carbonaceous material is an effective way to enhance the capacitance and lifespan of ZnFe_2_O_4_ for supercapacitors [21,22,23,24]. Due to the superior electrical conductivity and large theoretical specific surface area, graphene is the perfect candidate for dispersed nanoparticles. Therefore, designing composites by spreading binary transition metal oxides onto a graphene surface is feasible to improve electrode performance. Li et al. prepared ZnFe_2_O_4_ and nitrogen-doped graphene composites via the solvothermal method, which delivered specific capacitances of 244 F g^−1^ at a current density of 0.5 A g^−1^: much higher than pure ZnFe_2_O_4_ nanoparticles [25].

Furthermore, the material morphology and the particle size also have an important influence on the electrochemical properties of supercapacitors [26,27,28]. For instance, when a size-controllable ZnFe_2_O_4_/rGO hybrid was prepared through a solvothermal process, the high-performance supercapacitor electrode was achieved by controlling the ZnFe_2_O_4_ particle size [16]. In addition, a pure ZnFe_2_O_4_ deposited on nickel foam produced a specific capacitance of 552 F g^−1^ at a scan rate of 5 mV s^−1^ as a binder-free supercapacitor material [29]. Compared with the traditional heating process, microwave hydrothermal is a uniform heating process that occurs within the material, and it can achieve the synthesis of nano-sized particles within a few minutes [30,31,32,33]. Some researchers have made a great deal of progress in synthesizing ZnFe_2_O_4_ using the microwave hydrothermal method. For example, Wang et al. synthesized ZnFe_2_O_4_/rGO nanomaterials for the removal of methylene blue contamination by using a microwave hydrothermal technique, and achieved successful removal (contamination removal rate close to 100%) [34]. However, there is still limited research on using this facile microwave hydrothermal process to synthesize ZnFe_2_O_4_/rGO nanocomposites for supercapacitor electrodes.

Considering the above information, ZnFe_2_O_4_/rGO nanocomposites with nano-sized ZnFe_2_O_4_ were prepared using this facile microwave hydrothermal method. The generation of ZnFe_2_O_4_ nanoparticles and the reduction of GO were accomplished simultaneously. The effects of rGO content on morphology, chemical, and crystalline structure were investigated by powder X-ray diffraction (XRD), field-emission scanning electron microscope (FE-SEM), transmission electron microscopy (TEM), and high-resolution TEM (HR-TEM) tests. In addition, the electrochemical performance of ZnFe_2_O_4_/rGO nanocomposites with different rGO contents was evaluated with a series of electrochemical tests. We believe that this work can provide a new perspective for designing high-performance electrode materials for supercapacitors.

## 2. Materials and Methods

### 2.1. Materials

Tianjin Basf Chemical Co., Ltd. (Tianjin, China) was contacted for the procurement of graphite and acetylene black. The sodium nitrate (NaNO_3_), zinc chloride (ZnCl_2_), ferric chloride hexahydrate (FeCl_3_·6H_2_O), sodium hydroxide (NaOH), potassium permanganate (KMnO_4_), and hydrogen peroxide (H_2_O_2_) were purchased from Shanghai Sinopharm Co., Ltd. (Shanghai, China).

### 2.2. Materials Synthesis

The modified Hummers’ method was used to prepare graphene oxide (GO). The microwave hydrothermal process was selected to synthesize ZnFe_2_O_4_/rGO nanocomposites. First, 50 mg freeze-dried GO powder was dissolved in 50 mL distilled water and sonicated for 1 h to obtain a homogeneous suspension. Subsequently, ZnCl_2_ and FeCl_3_·6H_2_O were added into the GO suspension with a molar ratio of 1:2 and stirred for another 1 h. The pH value of the solution was then adjusted to 8 with the addition of NaOH (1 mol L^−1^). Next, the mixture was transferred into a sealed reactor with microwave heating for 7 min at a microwave power of 700 W and microwave frequency of 2450 MHz. Finally, the reactor was cooled down to room temperature naturally. To obtain the final product, the obtained solution was washed with ethanol and distilled water several times and freeze-dried for 40 h (denoted as ZnFe_2_O_4_/rGO−2). In addition, ZnFe_2_O_4_ was prepared under the same conditions without the addition of GO, and ZnFe_2_O_4_/rGO−1 and ZnFe_2_O_4_/rGO−3 were prepared by adjusting GO additions for comparison.

### 2.3. Materials Characterization

Powder X-ray diffraction (XRD) experiments using a Rigaku Smart-lab diffractometer equipped with Cu-Kα radiation (λ = 0.15418) from 20° to 70° at a scan rate of 10°/min were used to examine the crystal structures. A Thermo Scientific K-Alpha electron spectrometer was used for the X-ray photoelectron spectrometer (XPS) tests. A confocal laser micro-Raman spectrometer (LABRAMHR, JY Co., Parise, France) was used to record the Raman spectra. The morphology and microstructure of the obtained materials were examined on a field-emission scanning electron microscope equipped with an energy-dispersive X-ray spectrometer (FE-SEM, Zeiss Sigma 500). Transmission electron microscopy (TEM) and high-resolution TEM (HRTEM) were performed on a JSM-2100Plus (JEOL, Tokyo, Japan). On TG/DTA7200, thermogravimetric analysis (TGA) was carried out under an air atmosphere from 20 °C to 800 °C with a heating rate of 10 °C/min. The N_2_ adsorption and desorption isotherm was measured at 77 K on a Quantachrome ASiQwin AUTOSORB IQ device. The Brunaure Emmett Teller (BET) model and Barrett Joyner Halenda (BJH) model were used to analyze the specific surface area and pore size distribution. 

### 2.4. Electrochemical Measurements

Active materials (80 wt%), carbon black (10 wt%), and polytetrafluoroethylene (10 wt%) were mixed to prepare the slurry. The obtained slurry was coated with nickel foam with dimensions of 1 cm × 1 cm evenly. The foam was dried in a vacuum drying oven at 60 °C overnight, and then pressed at 10 MPa to obtain the working electrode. The loading of active materials for each electrode was around 0.8 mg. The three-electrode system was used to conduct electrochemical measurements at room temperature in a 2 mol L^−1^ KOH aqueous electrolyte. The platinum electrode and Hg/HgO electrode were used as the counter electrode and the reference electrode, respectively. Cyclic voltammetry (CV), galvanostatic charge/discharge (GCD), and electrochemical impedance spectroscopy (EIS) were performed on an electrochemical workstation (Zennium Pro, Zahner, Kronach Germany). EIS was carried out with an AC amplitude of 5 mV in a frequency range of 10 mHz to 100 KHz. The cycling performance of ZnFe_2_O_4_/rGO−2 was performed on the LAND battery test system (CT2001A).

## 3. Results and Discussion

**Figure 1:**
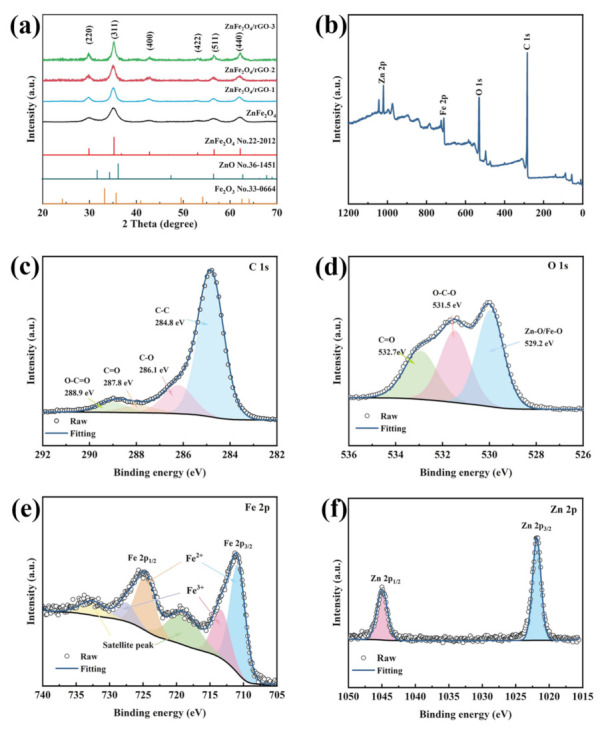
(**a**) XRD profiles of ZnFe_2_O_4_, ZnFe_2_O_4_/rGO–x (x = 1, 2, 3). Full XPS survey spectrum (**b**), High-resolution XPS spectrum of (**c**) C 1s, (**d**) O 1s, (**e**) Fe 2p, and (**f**) Zn 2p of ZnFe_2_O_4_/rGO−2.

The crystal structure and particle size of as-prepared ZnFe_2_O_4_ and ZnFe_2_O_4_/rGO nanocomposites were analyzed by X-ray diffraction (XRD). As Figure 1a shows, the cubic ZnFe_2_O_4_ (JCPDS No. 22-2012) lattice planes (220), (311), (400), (422), (511), and (440) were detected at the peaks of 29.9°, 35.3°, 42.8°, 53.1°, 56.6°, and 62.2°, respectively. No other peaks were found, which proves that this facile microwave hydrothermal process synthesized ZnFe_2_O_4_ with high purity. It is worth noting that the peak belonging to rGO disappeared, which can be explained by the highly crystalline nature of the ZnFe_2_O_4_ nanoparticles [35]. Moreover, in order to determine the particle size, the lateral crystal size (D) of the (311) plane of ZnFe_2_O_4_ was calculated by Scherrer’s equation [36]:

$$D=\frac{K\lambda }{\beta \sin\theta }$$

where K is the Scherrer constant equal to 0.9; λ = 1.5418 Å; β is the full width at half maximum (FWHM) of the diffraction peak; and θ is the corresponding Bragg angle of the (311) plane. The particle size was calculated to be 7.4, 7.6, 7.2, and 6.5 nm for ZnFe_2_O_4_, ZnFe_2_O_4_/rGO−1, ZnFe_2_O_4_/rGO−2, and ZnFe_2_O_4_/rGO−3, respectively. These results indicate that the nano-sized particles in pure ZnFe_2_O_4_ and ZnFe_2_O_4_/rGO nanocomposites were successfully synthesized by this facile microwave hydrothermal process.

### 3.1. Structure Analysis Morphology Analysis

X-ray photoelectron spectroscopy (XPS) characterization was carried out to investigate the surface compositions and chemical valence states of ZnFe_2_O_4_/rGO−2 nanocomposites. The full XPS survey spectrum (shown in Figure 1b) reveals the presence of C, O, Fe, and Zn elements, where the C elements came from rGO and the O, Fe, and Zn elements came from ZnFe_2_O_4_. As Figure 1c shows, the high-resolution XPS survey of C 1s was separated into four peaks at 284.4, 286.1, 287.8, and 288.9 eV, corresponding to C-C, C-O, C=O, and O-C=O bonds, respectively. The binding energy of O-Fe/O-Zn, O-C-C, and C=O were identified at 529.2, 531.5, and 532.7 eV, respectively (Figure 1d). The high-resolution XPS of Fe 2p demonstrated peaks corresponding to the two spin-orbital doublets, Fe 2p_3/2_ and Fe 2p_1/2_, along with the two shake-up satellite peaks for Fe (Figure 1e). The fitting peaks at 712.6 and 727.5 eV are ascribed to the Fe^3+^ state. The fitting peaks at 711.1 and 724.7 eV are related to the Fe^2+^ state. This confirmed that Fe^2+^ and Fe^3+^ were present together in ZnFe_2_O_4_/rGO−2 nanocomposites. The high-resolution XPS of the Zn 2p spectrum displays two characteristic peaks at 1021.8 and 1044.9 eV, corresponding to the spin orbits Zn 2p_3/2_ and Zn 2p_1/2_ of ZnFe_2_O_4_ (Figure 1f) [37,38].

**Figure 2:**
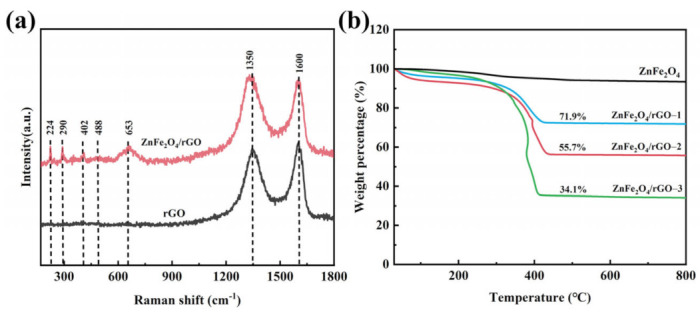
(**a**) Raman spectra of rGO and ZnFe_2_O_4_/rGO−2. (**b**) TGA curves of ZnFe_2_O_4_, ZnFe_2_O_4_/rGO−1, ZnFe_2_O_4_/rGO−2, and ZnFe_2_O_4_/rGO−3.

The local structures of the ZnFe_2_O_4_/rGO−2 nanocomposite and rGO were analyzed through a Raman spectra characterization (Figure 2a). Two obvious peaks can be observed at 1350 and 1600 cm^−1^, corresponding to the D and G bands of polycrystalline carbon materials. The D band corresponds to the defects in the atomic lattice, and the G band can be explained by the stretching of in-plane vibration of C-atom sp^2^ hybridization [38,39]. Hence, the defect level of carbon materials can be indicated by the ratio of the D and G bands (I_D_/I_G_). The ZnFe_2_O_4_/rGO−2 nanocomposite displayed a higher I_D_/I_G_ ratio (1.05) than pure rGO (0.91), indicating a more defective structure and a lower degree of graphitization created due to the presence of ZnFe_2_O_4_ nanoparticles [35]. Furthermore, in the Raman spectrum of ZnFe_2_O_4_/rGO−2, five peaks were detected at 224, 290, 402, 488, and 653 cm^−1^, corresponding to the vibration modes (A_1g_, E_g,_ and 3F_2g_) of ZnFe_2_O_4_. The movement pattern of oxygen in the tetrahedral AO_4_ group corresponds to the mode at 653 cm^−1^ (A_1g_). Other patterns in the low-frequency region represent the characteristics of octahedrons, where the mode at 290 cm^−1^ corresponds to E_g_, and the modes at 224, 402, and 488 cm^−1^ correspond to F_2g_.

Thermogravimetric analysis (TGA) was carried out on all samples to obtain the weight percentage of rGO in ZnFe_2_O_4_/rGO composites (Figure 2b). Due to the existence of adsorbed water and the residual oxygen functional groups of rGO, all samples exhibited mass loss under 150 °C. In addition, the dominant mass loss of all ZnFe_2_O_4_/rGO nanocomposites between 300 to 400 °C was attributed to the burning of rGO. After fully burning, the residual was ZnFe_2_O_4_. Hence, the calculated mass percentages of rGO in ZnFe_2_O_4_/rGO−1, ZnFe_2_O_4_/rGO−2, and ZnFe_2_O_4_/rGO−3 were 28.1%, 44.3% and 65.9%, respectively.

**Figure 3:**
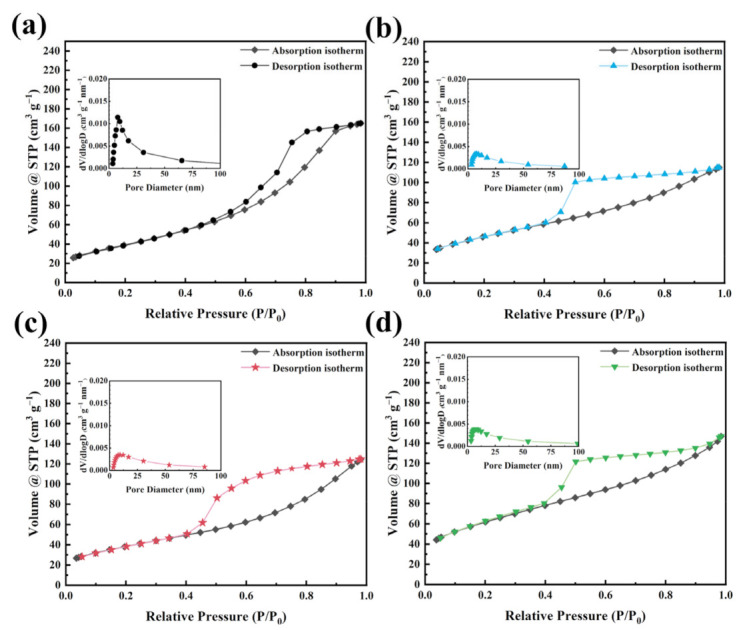
N_2_ adsorption-desorption isotherms and pore size distribution of (**a**) ZnFe_2_O_4_, (**b**) ZnFe_2_O_4_/rGO−1, (**c**) ZnFe_2_O_4_/rGO−2, and (**d**) ZnFe_2_O_4_/rGO−3.

The specific surface area and pore size distribution of the ZnFe_2_O_4_/rGO nanocomposites were investigated by N_2_ adsorption and desorption experiments, and the results are shown in Figure 3. All of the as-prepared samples exhibited simple type IV hysteresis IUPAC lines, indicating the existence of mesoporous structure. The specific surface area of ZnFe_2_O_4_/rGO−1, ZnFe_2_O_4_/rGO−2, and ZnFe_2_O_4_/rGO−3 were calculated to be 150, 180, and 170 m^2^ g^−1^, respectively. This demonstrates that the addition of rGO has effects on the specific surface area of the sample. The ZnFe_2_O_4_ nanoparticles interspersed on the graphene sheets form more pore structures, and the pore size distribution is shown in Figure 3a through Figure 3d. According to the Barrett Joyner Halenda (BJH) model, the average pore diameters of ZnFe_2_O_4_/rGO−1, ZnFe_2_O_4_/rGO−2, and ZnFe_2_O_4_/rGO−3 were 4.11 nm, 5.59 nm, and 4.28 nm, respectively. The highest specific surface area and optimal pore size distribution can increase the electrochemically active sites for redox reactions and provide ionic diffusion channels to enhance ion transportation.

### 3.2. Morphology Analysis

**Figure 4:**
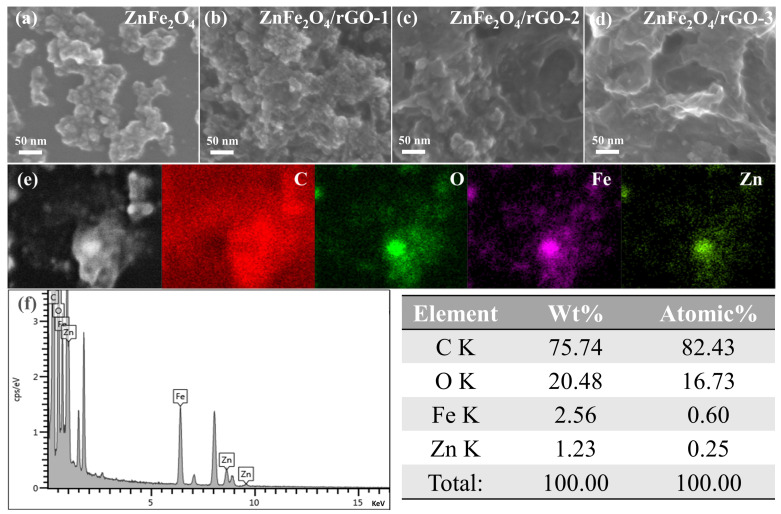
SEM images of (**a**) ZnFe_2_O_4_, (**b**) ZnFe_2_O_3_/rGO−1, (**c**) ZnFe_2_O_3_/rGO−2, and (**d**) ZnFe_2_O_3_/rGO−3, (**e**) SEM elemental mapping, and (**f**) elemental content of ZnFe_2_O_4_/rGO−2 nanocomposites.

A field-emission scanning electron microscope (FE-SEM) was used to characterize the detailed morphology and microstructure of ZnFe_2_O_4_ and ZnFe_2_O_4_/rGO nanocomposites. Figure 4a shows the serious aggregation of ZnFe_2_O_4_ nanoparticles prepared without GO addition. When less GO was added, Figure 4b exhibits that the aggregation of ZnFe_2_O_4_ nanoparticles was partially improved. The ZnFe_2_O_4_/rGO−2 (Figure 4c) with medium content of rGO shows that the ZnFe_2_O_4_ nanoparticles were uniformly anchored onto the rGO surface without obvious aggregation. However, when the amount of GO continues to increase (Figure 4d), the nanoparticles cannot fully occupy the surface of rGO and the large specific surface area of rGO cannot be effectively utilized. From the FE-SEM elemental mapping images and elemental content of ZnFe_2_O_4_/rGO−2 nanocomposites (Figure 4e), the element distribution of C, O, Fe, and Zn can be observed. Figure 4f shows the elemental contents of ZnFe_2_O_4_/rGO−2 at the selected area.

**Figure 5:**
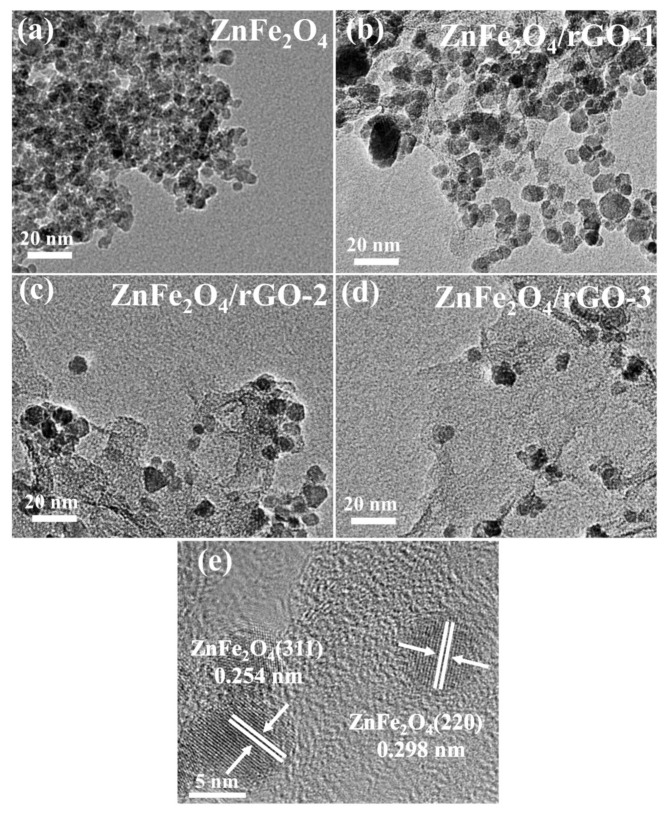
TEM images of (**a**) pure ZnFe_2_O_4_, (**b**) ZnFe_2_O_4_/rGO−1, (**c**) ZnFe_2_O_4_/rGO−2, and (**d**) ZnFe_2_O_4_/rGO−3. (**e**) HRTEM of ZnFe_2_O_4_/rGO−2.

Transmission electron microscopy (TEM) and high-resolution TEM (HRTEM) characterization were utilized to investigate detailed microstructure features of pure ZnFe_2_O_4_ and ZnFe_2_O_4_/rGO nanocomposites (results shown in Figure 5). The TEM pictures showed the same results as the SEM pictures, wherein the aggregation of ZnFe_2_O_4_ nanoparticles was reduced as the content of rGO increased. As shown in Figure 5c, the particle size of ZnFe_2_O_4_ nanoparticles in ZnFe_2_O_4_/rGO−2 was approximately 8 nm, which is close to the XRD results. Figure 5e shows the HRTEM of ZnFe_2_O_4_/rGO−2. The lattice spacing of ZnFe_2_O_4_ was 0.254 nm and 0.298 nm, corresponding to the spacing of the (311) and (220) crystalline planes, which indicates the same results as XRD (Figure 1a).

### 3.3. Electrochemical Performance of Supercapacitor

**Figure 6:**
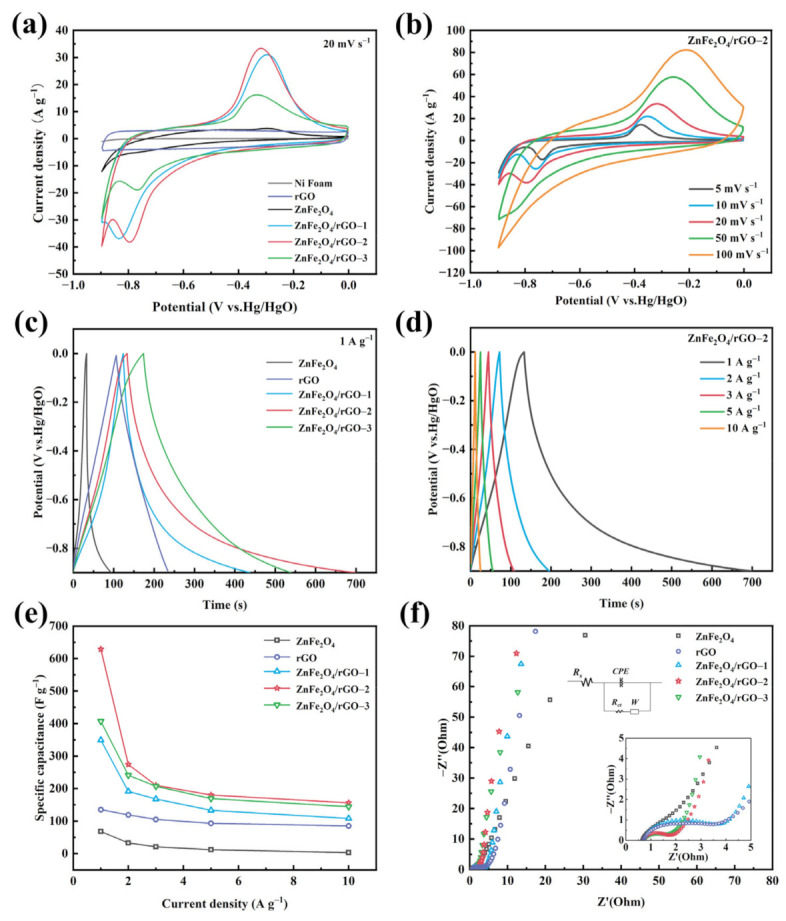
(**a**) CV curves of ZnFe_2_O_4_, rGO, ZnFe_2_O_4_/rGO−X (X = 1, 2, 3) at 20 mV s^−1^ and (**b**) ZnFe_2_O_4_/rGO−2 at different scan rate, (**c**) charge/discharge curves of the ZnFe_2_O_4_, rGO, ZnFe_2_O_4_/rGO−X (X = 1, 2, 3) at 1 A g^−1^ and (**d**) ZnFe_2_O_4_/rGO−2 at 1 A g^−1^, (**e**) rate performance and (**f**) Nyquist plots of ZnFe_2_O_4_, rGO, ZnFe_2_O_4_/rGO−X (X = 1, 2, 3).

For comparison, reduced graphene oxide(rGO) was prepared using the same method as described above, and the electrochemical properties of ZnFe_2_O_4_, rGO, and ZnFe_2_O_4_/rGO−X (X = 1, 2, 3) were explored. Figure 6a shows the CV curves of ZnFe_2_O_4_, rGO, and ZnFe_2_O_4_/rGO nanocomposites at 20 mV s^−1^. An approximately rectangular shape can be seen on the CV curve of rGO, which is a common electric double-layer capacitance characteristic. The ZnFe_2_O_4_ and ZnFe_2_O_4_/rGO nanocomposites showed obvious redox peaks, which can be explained that by the predominance of the pseudocapacitance contribution. In addition, the ZnFe_2_O_4_/rGO−2 shows the biggest area among all of the ZnFe_2_O_4_ and ZnFe_2_O_4_/rGO nanocomposites, indicating the highest specific capacitance. This can be attributed to the synergistic interaction between ZnFe_2_O_4_ and rGO, where ZnFe_2_O_4_ inhibits rGO self-stacking and rGO also effectively restrains ZnFe_2_O_4_ agglomeration, thereby supplying more active sites for the electrochemical process, increasing the electrochemical performance and charge storage capacity. The CV curves of the ZnFe_2_O_4_/rGO−2 composites electrode are shown in Figure 6b with scan rates from 5 to 100 mV s^−1^ at a potential window of −0.9 to 0 V. The oxidation peaks and reduction peaks during the redox reaction can be explained by the following equation [40]:

$${\mathrm{ZnFe}}_{2}O_{4}+2e^{-}+3H_{2}O\;⇌\;\mathrm{ZnO}\;+2\mathrm{Fe}{\left(\mathrm{OH}\right)}_{2}+2{\mathrm{OH}}^{-}$$

Figure 6c shows the charge and discharge profiles of ZnFe_2_O_4_, rGO, and ZnFe_2_O_4_/rGO nanocomposites at 1 A g^−1^. According to the following equation, the longest discharge time indicates the highest specific capacity, hence the ZnFe_2_O_4_/rGO−2 nanocomposites possess the highest specific capacity, which is the same as the CV results:

$$\mathrm{Cs}=\frac{I\Delta t}{m\Delta V}$$

where C_s_ is the specific capacitance (F g^−1^), I is the discharge current (A), Δt is the discharge time (s), m is the mass of active materials (g) and ΔV is the voltage change during the discharging process (V). Figure 6d shows the GCD curves of ZnFe_2_O_4_/rGO−2 at different current densities. The specific capacitance of ZnFe_2_O_4_/rGO−2 was calculated to be 628 F g^−1^ at 1 A g^−1^, and it can remain at 156 F g^−1^ when the current density increases to 10 A g^−1^, which is higher than all of the ZnFe_2_O_4_, rGO, ZnFe_2_O_4_/rGO−1, and ZnFe_2_O_4_/rGO−3 nanocomposites (Figure 6e). The apparent decrease of specific capacitance of all samples can be attributed to the fact that as the current density increases, the electrode-electrolyte interface absorbs a large number of electrolyte ions, which leads to a rapid drop in the electrolyte ion concentration at, or near, the interface, combined with the increase of electrode polarization. The electrochemical performance of ZnFe_2_O_4_/rGO is closely related to the rGO content. When the rGO content is low (ZnFe_2_O_4_/rGO−1), there are only a few electrochemically active sites, and the ion diffusion is restricted due to the agglomeration of ZnFe_2_O_4_ during the electrochemical process. Conversely, fewer redox reactions occur when the reduced graphene oxide content is higher (ZnFe_2_O_4_/rGO−3), owing to the relatively low content of ZnFe_2_O_4_.

In addition, we calculated the specific capacitance according to the cyclic voltammetry test results, according to the following formula:

$$\mathrm{Cs}=\int_{V1}^{V2}\frac{\mathrm{idV}}{2\mathrm{mv}\Delta V}$$

where Cs is the specific capacitance (F g^−1^), i is the corresponding current (mA), V_1_ and V_2_ are the two working potential limits (V), m is the mass of active materials (g), ΔV is the voltage window (V), and v is the scan rate (mV s^−1^). According to the calculated results (Figure S1), in comparison to rGO and ZnFe_2_O_4_, ZnFe_2_O_4_/rGO composites exhibit high specific capacitance. It is worth mentioning that ZnFe_2_O_4_/rGO−2 has a specific capacitance of 554 F g^−1^ at 5 mV s^−1^, maintains a specific capacitance of 261 F g^−1^ at 100 mV s^−1^, and has a specific capacitance of 628 F g^−1^ at 1 A g^−1^. ZnFe_2_O_4_/rGO−2 is assumed to have the best performance since it has the highest specific surface area, the best pore size distribution, a uniform ZnFe_2_O_4_ dispersion, and the fastest redox reaction on the surface even when subjected to high currents.

The essential performance of ZnFe_2_O_4_, rGO, and ZnFe_2_O_4_/rGO−X (X = 1, 2, 3) nanocomposite electrodes were further explored with Nyquist plots. The Nyquist plots can be divided into three parts. In the low-frequency region, the line corresponds to the diffusion impedance. Thus, a large slope indicates a faster diffusion capability. The semi-circle existing in the medium-frequency region corresponds to charge transfer resistance (Rct) and the intercept of the plot with the Z′-axis in the high-frequency region indicates the contact resistance (Rc). As shown in the inset of Figure 6f, the Nyquist plots of ZnFe_2_O_4_/rGO are a semicircle in the high-frequency region and straight in the low-frequency region. The data of the electrochemical impedance spectroscopy is fitted by the equivalent resistance plot. The ZnFe_2_O_4_/rGO−2 electrode shows intrinsic resistance (Rs) and charge transfer resistance (Rct) 0.73 Ω and 0.84 Ω. The ZnFe_2_O_4_/rGO−2 exhibits the smallest charge transfer resistance, comparable intrinsic resistance, and good diffusion capability among ZnFe_2_O_4_ and ZnFe_2_O_4_/rGO composites. The largest specific capacitance of ZnFe_2_O_4_/rGO−2 can be attributed to its improved diffusion and high conductivity.

**Figure 7:**
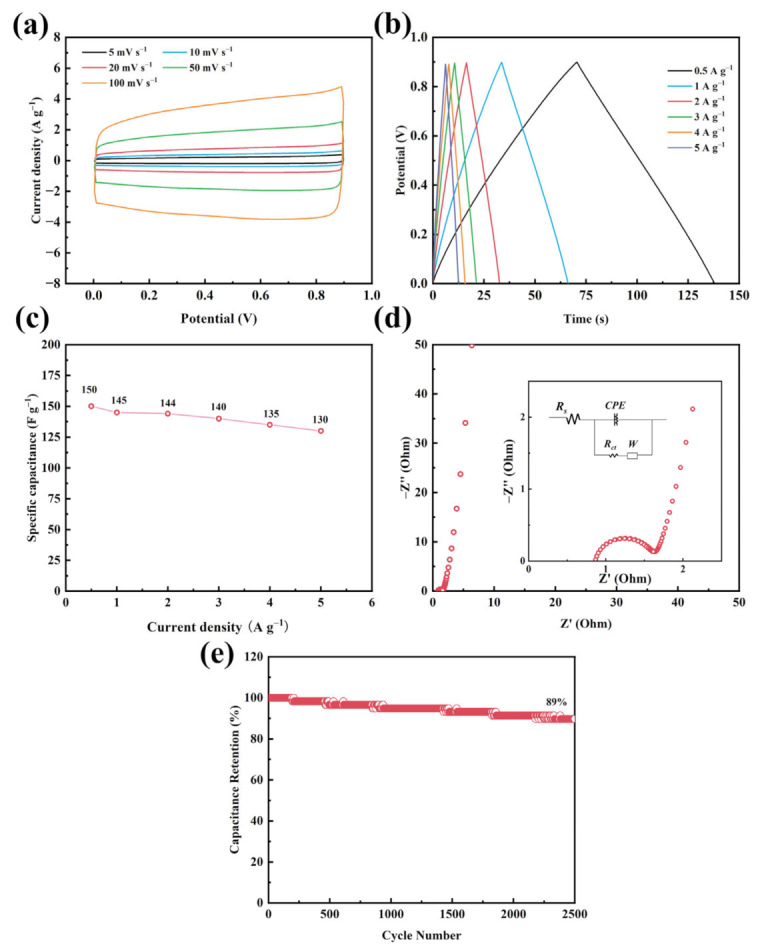
(**a**) CV curves, (**b**) charge/discharge profiles, (**c**) rate performance, (**d**) Nyquist plots and, (**e**) cycle stability tests at 1 A g^−1^ of ZnFe_2_O_4_/rGO−2 for symmetrical supercapacitor.

An asymmetrical supercapacitor was assembled using ZnFe_2_O_4_/rGO−2 electrodes, 2M KOH as the electrolyte, and glass fiber (GF/D) as the separator. Figure 7a shows the CV curves of symmetric supercapacitor at various scan rates from 0 to 0.9 V. There is no obvious redox peak that closely resembles the rectangles. The CV curve remained fairly rectangular in shape without significantly changing as the scan rate was raised from 5 mV s^−1^ to 100 mV s^−1^. Figure 7b shows plots of constant current charge and discharge at various current densities. The triangular charge and discharge curves show good capacitive performance. Figure 7c shows the rate performance of ZnFe_2_O_4_/rGO−2 for a symmetrical supercapacitor. The specific capacitance of 150 F g^−1^ was achieved at 0.5 A g^−1^, which can still maintain 86.7% at 5 A g^−1^. The equivalent resistance plot, which represents the electrochemical AC impedance of a symmetrical supercapacitor, exhibits intrinsic resistance (Rs) and charge transfer resistance (Rct) 0.91 Ω and 0.57 Ω (Figure 7d). Cyclic stability is a crucial consideration for a candidate electrode material for the supercapacitor’s practical application. In Figure 7e, the capacitance retention rate of ZnFe_2_O_4_/rGO−2 is 89% after 2500 cycles at 1 A g^−1^. Table 1 exhibits the higher or comparable electrochemical performance of ZnFe_2_O_4_/rGO−2, indicating the advantages of this facile microwave hydrothermal process. Furthermore, when compared with previous reports, our work also shows superior or comparable specific capacitance. For exploring the high conductivity of rGO, the comparison of pure rGO and carbon black/rGO was provided. As shown in Figure S2, pure rGO shows the nearly similar electrochemical performance to carbon black/rGO, the results indicate the he high conductivity of synthesized rGO by microwave hydrothermal process.

## 4. Conclusions

In conclusion, a facile microwave hydrothermal process was applied to synthesize ZnFe_2_O_4_/rGO nanocomposites. The as-prepared ZnFe_2_O_4_/rGO nanocomposites showed uniform distribution of ZnFe_2_O_4_ less than 10 nm on the rGO surface, and higher specific capacitance and rich pore structure. The ZnFe_2_O_4_/rGO nanocomposites with 44.3 wt.% rGO show the highest specific capacitance (628 F g^−1^ at 1 A g^−1^), which is much higher than the pure ZnFe_2_O_4_ (68 F g^−1^ at 1 A g^−1^). When ZnFe_2_O_4_/rGO−2 was prepared as the electrode of the symmetric supercapacitor, it exhibited a capacitance retention of 89% for 2500 cycles. These results demonstrate that this facile microwave hydrothermal process could be used in preparing other binary transition metal oxide and carbon material nanocomposites for supercapacitors electrodes.

## Figures and Tables

**Table 1:** The comparison with previously reported ZnFe_2_O_4_-based supercapacitor materials.

Materials	Specific Capacitance	Cycle Performance	Reference
Active Carbon/MWCNTs@ZnFe_2_O_4_	5 mV s^−1^ 613F g^−1^	91% at 1 A g^−1^ after 10,000 cycles	[23]
ZnFe_2_O_4_/rGO	1 A g^−1^ 352.9 F g^−1^	76.5% at 10 A g^−1^ after 10,000 cycles	[16]
ZnFe_2_O_4_/N-rGO	0.5 A g^−1^ 352.9 F g^−1^	83.8% at 100 mV s^−1^ after 5000 cycles	[25]
ZnFe_2_O_4_/rGO	0.5 A g^−1^ 314 F g^−1^	77.6% at 5 A g^−1^ after 1500 cycle/s	[20]
ZnFe_2_O_4_/rGO	1 A g^−1^ 628 F g^−1^	89% at 1 A g^−1^ after 2500 cycles	This work

## Data Availability

Data is contained within the article.

## Supplementary Materials

The following supporting information can be downloaded at: https://www.mdpi.com/article/10.3390/nano13061034/s1, Figure S1: Plot of Scanning rate and specific capacitance from 5 mV s^−1^ to 100 mV s^−1^, Figure S2: (**a**) CV curves and (**b**) Charge/discharge profiles of carbon black/rGO, (**c**) CV curves and (**d**) Charge/discharge curves of carbon black/rGO of pure rGO, (**e**) Rate performance and (**f**) Nyquist plots of carbon black/rGO and pure rGO.
