# Supplementary material for: A Facile Microwave Hydrothermal Synthesis of ZnFe2O4/rGO Nanocomposites for Supercapacitor Electrodes
Source: Nanomaterials (Basel). 2023 Mar 13;13(6):1034. doi: 10.3390/nano13061034 (PMC10053183; doi:10.3390/nano13061034)
Supplement: Supplementary file 1 [file nanomaterials-13-01034-s001.zip › nanomaterials-2207881-supplementary.pdf]

Supplementary Materials

A Facile Microwave Hydrothermal Synthesis of $ZnFe_2O_4$/rGO Nanocomposites for Supercapacitor Electrodes

Xiaoyao Mo [1], Guangxu Xu [1], Xiaochan Kang [1], Hang Yin [1], Xiaochen Cui [2], Yuling Zhao [3], Jianmin Zhang [2], Jie Tang [4,*] and Fengyun Wang [1,*]

[1] College of Physics, Qingdao University, No. 308 Ningxia Road, Qingdao 266071, China
[2] College of Mechanical and Electrical Engineering, Qingdao University, No. 308 Ningxia Road, Qingdao 266071, China
[3] State Key Laboratory of Bio Fibers and Eco Textiles, Qingdao University, No. 308 Ningxia Road, Qingdao 266071, China
[4] National Institute for Materials Science, 1-2-1 Sengen, Tsukuba 305–0047, Japan
* Correspondence: author: tang.jie@nims.go.jp (J.T.), fywang@qdu.edu.cn (F.W.)

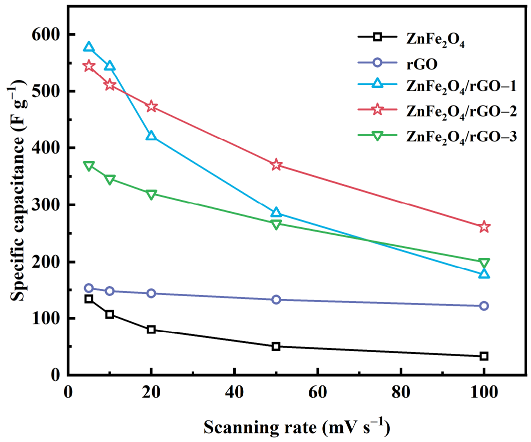

Figure S1. Plot of Scanning rate and specific capacitance from 5 mV s^{-1} to 100 mV s^{-1}.

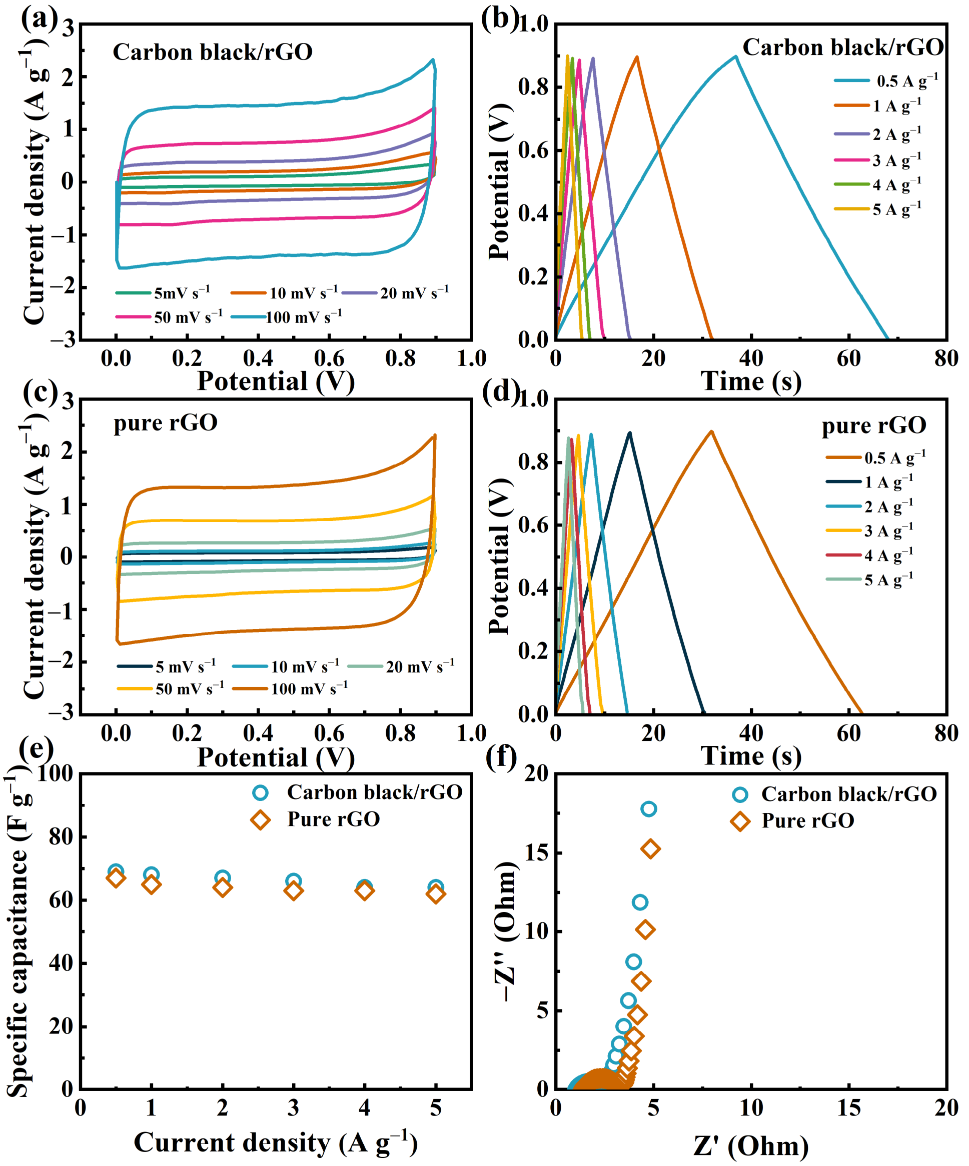

Figure S2. (**a**) CV curves and (**b**) Charge/discharge profiles of carbon black/rGO, (**c**) CV curves and (**d**) Charge/discharge curves of carbon black/rGO of pure rGO, (**e**) Rate performance and (**f**) Nyquist plots of carbon black/rGO and pure rGO.

Figure S2a, c exhibited the CV curves of carbon black/rGO and pure rGO from 5 mV s^{-1} to 100 mV s^{-1}. The typical rectangular of CV curves indicate an EDLC capacitive behavior during the charging/discharging process. Besides, symmetrical charging/discharging triangles with neglectable voltage drop in Figure S2b, d demonstrate the low internal resistances of the carbon black/rGO and pure rGO. According to the charge/discharge profiles at different current density, we calculated the specific capacitance of carbon black/rGO and pure rGO, the results were shown in Fig, S2e. The comparable rate performance and Nyquist plots (Figure S2f) of carbon black/rGO and pure rGO exhibited the high conductivity of synthesized rGO by microwave hydrothermal process.
